# Corneal and Epidermal Nerve Quantification in Chemotherapy Induced Peripheral Neuropathy

**DOI:** 10.3389/fmed.2022.832344

**Published:** 2022-02-18

**Authors:** Nilo Riva, Filippo Bonelli, Romina Mayra Lasagni Vitar, Marco Barbariga, Philippe Fonteyne, Ignazio Diego Lopez, Teuta Domi, Fabio Scarpa, Alfredo Ruggeri, Michele Reni, Magda Marcatti, Angelo Quattrini, Federica Agosta, Paolo Rama, Giulio Ferrari

**Affiliations:** ^1^Experimental Neuropathology Unit, Division of Neuroscience, Institute of Experimental Neurology (INSPE), IRCCS San Raffaele Scientific Institute, Milan, Italy; ^2^Neurology and Neurorehabilitation Unit, Istituto di Ricovero e Cura a Carattere Scientifico (IRCCS) San Raffaele Scientific Institute, Milan, Italy; ^3^Cornea and Ocular Surface Disease Unit, Eye Repair Laboratory, IRCCS San Raffaele Scientific Institute, Milan, Italy; ^4^Department of Information Engineering, University of Padua, Padua, Italy; ^5^Resono Ophthalmic srl, Trieste, Italy; ^6^Department of Oncology, Medical Oncology Unit, San Raffaele Scientific Institute, Milan, Italy; ^7^Hematology and Bone Marrow Transplantation Unit, Department of Oncohematology, San Raffaele Scientific Institute, Milan, Italy; ^8^Vita-Salute San Raffaele University, Milan, Italy; ^9^Neuroimaging Research Unit, Institute of Experimental Neurology (INSPE), Division of Neuroscience, IRCCS San Raffaele Scientific Institute, Milan, Italy

**Keywords:** corneal confocal microscopy, chemotherapy-induced neuropathy, skin biopsy, nerves, neurotoxicity, cornea

## Abstract

Chemotherapy-induced neurotoxicity is an increasingly recognized clinical issue in oncology. *in vivo* confocal microscopy (IVCM) of corneal nerves has been successfully used to diagnose peripheral neuropathies, including diabetic neuropathy. The purpose of this study was to test if the combination of corneal nerve density and morphology assessed by IVCM is useful to monitor the neurotoxic effects of chemotherapy compared to epidermal nerve quantification. Overall, 95 adult patients with different cancer types were recruited from the oncology and hematology departments of the San Raffaele Hospital. Neurological examination, including clinical Total Neuropathy Score, and *in vivo* corneal confocal microscopy (IVCM), were performed before and after chemotherapy. In a group of 14 patients, skin biopsy was performed at the first and last visit. In the group of 14 patients who underwent both skin biopsy and corneal nerve imaging, clinical worsening (+69%, *p* = 0.0018) was paralleled by corneal nerve fiber (CNF) density reduction (−22%, *p* = 0.0457). Clinical Total neuropathy score significantly worsened from the first to the last visit (+62%, *p* < 0.0001). CNF length was not significantly reduced overall. However, CNF density/tortuosity ratio significantly decreased after therapy. Correlation analysis showed that the CNF density/tortuosity ratio was also correlated with the number of chemotherapy cycles (*r* = −0.04790, *P* = 0.0009). Our data confirm that *in vivo* corneal confocal microscopy is a helpful, non-invasive tool which shows promise for the diagnosis of chemotherapy-induced peripheral neuropathies. IVCM could allow a rapid, reproducible and non-invasive quantification of peripheral nerve pathology in chemotherapy-associated neuropathy.

## Introduction

Chemotherapy-induced neurotoxicity is one of the most common side effects of cancer treatment. It has been recognized as a major determinant of functional impairment, increased pain, and reduced quality of life (1, 2). Its prevalence reaches 68% in the first month following chemotherapy, and persists after more than 6 months in one-third of them (2). Chemotherapeutic agents that are routinely used to treat breast, lung, ovarian, bladder, and colon cancers are associated with neurotoxicity. Paclitaxel, for instance, can induce mild to moderate neuropathy in 93% of treated patients, which progresses to severe in 25% of them (3–5). Likewise, platinum compounds lead to chemotherapy-induced peripheral neuropathy (CIPN) in up to 50% of the patients (6–8). CIPN has been reported also following Bortezomib, with a prevalence ranging from 38 to 53%, and after thalidomide, with a frequency of 63% (8, 9). Similarly, cyclophosphamide has been reported to have neurotoxic effects on both younger and elderly patients (7, 10).

The diagnosis of CIPN is currently based on clinical testing, supported by neurophysiological studies and/or skin biopsy to evaluate epidermal nerve density. Although skin biopsy is a minimally invasive technique, it is not well accepted by some patients and it is not an option when frequent and/or repeated evaluations are needed (11).

*In vivo* corneal confocal microscopy (IVCM) has been successfully used to diagnose and stage peripheral neuropathies -specifically diabetic- showing higher sensitivity than skin biopsy (12–14).

Proper assessment and early diagnosis of peripheral neuropathy is crucial to allow prompt treatment modulation or interruption in most severe cases (1). In this paper, we propose that the combination of corneal nerve density and morphology assessed by IVCM is useful to diagnose and monitor the neurotoxic effects of chemotherapy.

## Methods

### Patients

Ninety five patients, 63 males and 32 females, were enrolled for the study. The average age was 59 years, with the youngest patient being 31 and the oldest 82 years old. Inclusion criteria were as follows: age > 18 years; exposure to a regimen of neurotoxic chemotherapeutic agents (Paclitaxel, platinum compounds, Bortezomib-Thalidomide-Dexamethasone (VTD), or Cyclophosphamide-combined treatments); expected survival of at least 6 months. Exclusion criteria were: prior neurotoxic therapy; diabetes; pre-existing peripheral neuropathy; corneal disease/surgery; contact lens wearing or eye drop application. Patients who met inclusion/exclusion criteria and signed the informed consent were recruited from the Oncology and Hematology Departments of the San Raffaele Hospital between 2011 and 2016. The study was carried out in accordance with the guidelines established by the Declaration of Helsinki, and the Institutional Review Board/Ethics committee (Comitato Etico Istituto Scientifico Ospedale San Raffaele) approval was obtained.

### Clinical and Imaging Parameters

Neurological examination, including clinical Total Neuropathy Score (cTNS) (15) and IVCM evaluation, were performed before and within 4 weeks after completion of chemotherapy. Briefly, cTNS is a comprehensive parameter that includes signs, symptoms and objective testings. In particular, scores are attributed according to the patient's motor, sensory and autonomic abilities, and neurological signs of muscle power, reflexes and pain sensitivity are also evaluated. Then, the change in cTNS (ΔcTNS) observed after chemotherapy was calculated as follows: ΔcTNS = cTNS after – cTNS before.

Cumulative dose was obtained by multiplying the selected dose by the number of cycles received from the patients.

Corneal confocal images were taken using a Heidelberg Retina Tomograph III, equipped with the Rostock Cornea Module (Heidelberg Engineering, Heidelberg, Germany) as previously described (16). Corneal confocal images (total number: 14.716 images) were normalized and analyzed by applying the deep learning technique Convolutional Neural Network (CNN), which includes Gabor filtering to enhance nerve visibility (17). Corneal Nerve Fiber length was determined as cumulative length of the corneal nerves per unit of area of the cornea (mm/mm^2^). Corneal nerve tortuosity was calculated by using the short-range tortuosity algorithm, as previously described (18, 19). Short-range tortuosity is defined as frequent, small-amplitude directional changes in nerves (20, 21). A ratio was calculated, as previously described (22), by dividing CNF length with corneal nerve tortuosity, to have a more comprehensive vision on patient's parameters.

Skin biopsy was collected from the lateral-malleolar region 10 cm upper from the malleolus in 14 random-selected patients who signed the informed consent before and after treatment. Epidermal nerve fibers were counted manually, and density was obtained by dividing the number of nerve fibers over the epidermal length.

### Statistics

To test differences in nerve parameters before and after treatment, Mann-Whitney and Spearman's non-parametric tests were used for continuous and ordinal variables. One-sample *t*-test was used to compare ΔTNS among the experimental groups. Two-sided *p* < 0.05 was considered statistically significant for all comparisons. Results are presented as mean ± standard error of the mean (SEM). The statistical analysis was performed using GraphPad Prism 8.0 (GraphPad Software, La Jolla, CA).

## Results

### Clinical and IVCM Neuropathy Assessment

**Figure 1:**
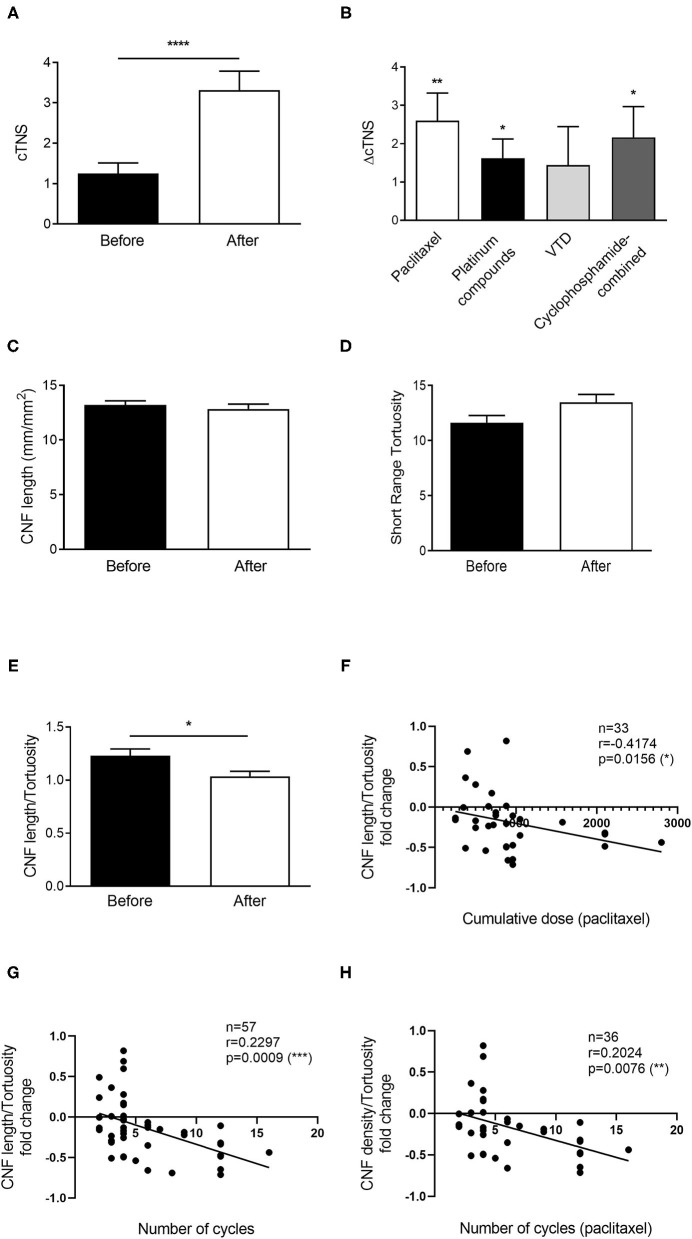
Chemotherapy-induced neuropathy is demonstrated by reduced CNF length/tortuosity index in a large cohort of patients. **(A)** Clinical TNS calculated in patients before and after therapy. **(B)** Average ΔTNS calculated before and after the different therapies. Paclitaxel *n* = 15 patients, Platinum compounds *n* = 8 patients, VTD *n* = 9 patients, Cyclophosphamide-combined *n* = 6 patients. **(C)** Corneal Nerve Fiber length before and after therapy. **(D)** Corneal nerve tortuosity obtained by using the short-range tortuosity algorithm before and after the therapy. **(E)** Corneal Nerve Fiber length—tortuosity ratio before and after therapy. **(F)** Scatter plot correlating the cumulative dose received from patients treated with paclitaxel with the corneal nerve fiber density/tortuosity ratio. **(G)** Scatter plot correlating the number of chemotherapy cycles with the corneal nerve fiber density/tortuosity ratio. **(H)** Scatter plot correlating the number of chemotherapy cycles of paclitaxel with the corneal nerve fiber density/tortuosity ratio. Histograms represent mean values ± SEM; Statistical analysis by Mann-Whitney non-parametric test, One-sample *t*-test or Spearman's Rank-Order Correlation (*p < 0.05, **p < 0.01, ***p < 0.001, ****p < 0.0001).

Ninety-five patients, including the 14 that underwent epidermal nerve examination, were recruited. Details of cancer types are listed in Table 1. Of them, 22 were lost at the post-treatment follow-up; therefore, 73 patients were considered for the analysis. Thirty two patients fully completed the cTNS examination before and after chemotherapy. All the patients received a complete corneal examination. In those patients, cTNS significantly worsened from the first to last visit (+62%, *p* < 0.0001, Figure 1A). cTNS worsened significantly after treatment with Paclitaxel (*p* = 0.0029), Platinum compounds (*p* = 0.0138), and in combination with Cyclophosphamide (*p* = 0.0428), while no significant difference was found in the VTD group (*p* = 0.1872, Figure 1B). CNF length (*p* = 0.57, Figure 1C) and corneal nerve short range tortuosity (*p* = 0.1350, Figure 1D) showed no significant difference. Intriguingly, however, combining the two parameters by calculating their ratio (CNF length/tortuosity), resulted in a significant decrease after chemotherapy (*p* = 0.0333, Figure 1E).

**Table 1:** Demographics of the large cohort of patients.

**Drug**	**Total number of patients**	**Mean age**	**Sex**	**Cancer types (number of patients)**
Paclitaxel	36	58	29 F, 7 M	Breast (22)
				Pancreatic (14)
Platin	12	66	6 F, 6 M	Colon (4)
				Breast (3)
				Pancreatic (2)
				Tonsil (1)
				Bladder (1)
				Multiple myeloma (1)
VTD	11	57	5 F, 6 M	Multiple myeloma (9)
				Amiloidosis (2)
Cyclophosphamide-combined	15	54	10 F, 5 M	Breast (7)
				Multiple myeloma (6)
				Amiloidosis (2)

Further analysis revealed that cumulative dose of patients receiving paclitaxel, which was the only group having an adequate number of patients, was correlated with CNF length/tortuosity reduction (*r* = −4174, *p* = 0.0156, Figure 1F). Similarly, the number of chemotherapy cycles was correlated with CNF length/tortuosity reduction (*r* = −0.04790, *p* = 0.0009, Figure 1G). Subgroup correlation analysis for paclitaxel confirmed the previous result, indicating that an increasing number of cycles corresponded to worse corneal parameters (*r* = 0.2024, *p* = 0.0076, Figure 1H).

### Dissection of Corneal Parameters in Single Drug Groups

**Figure 2:**
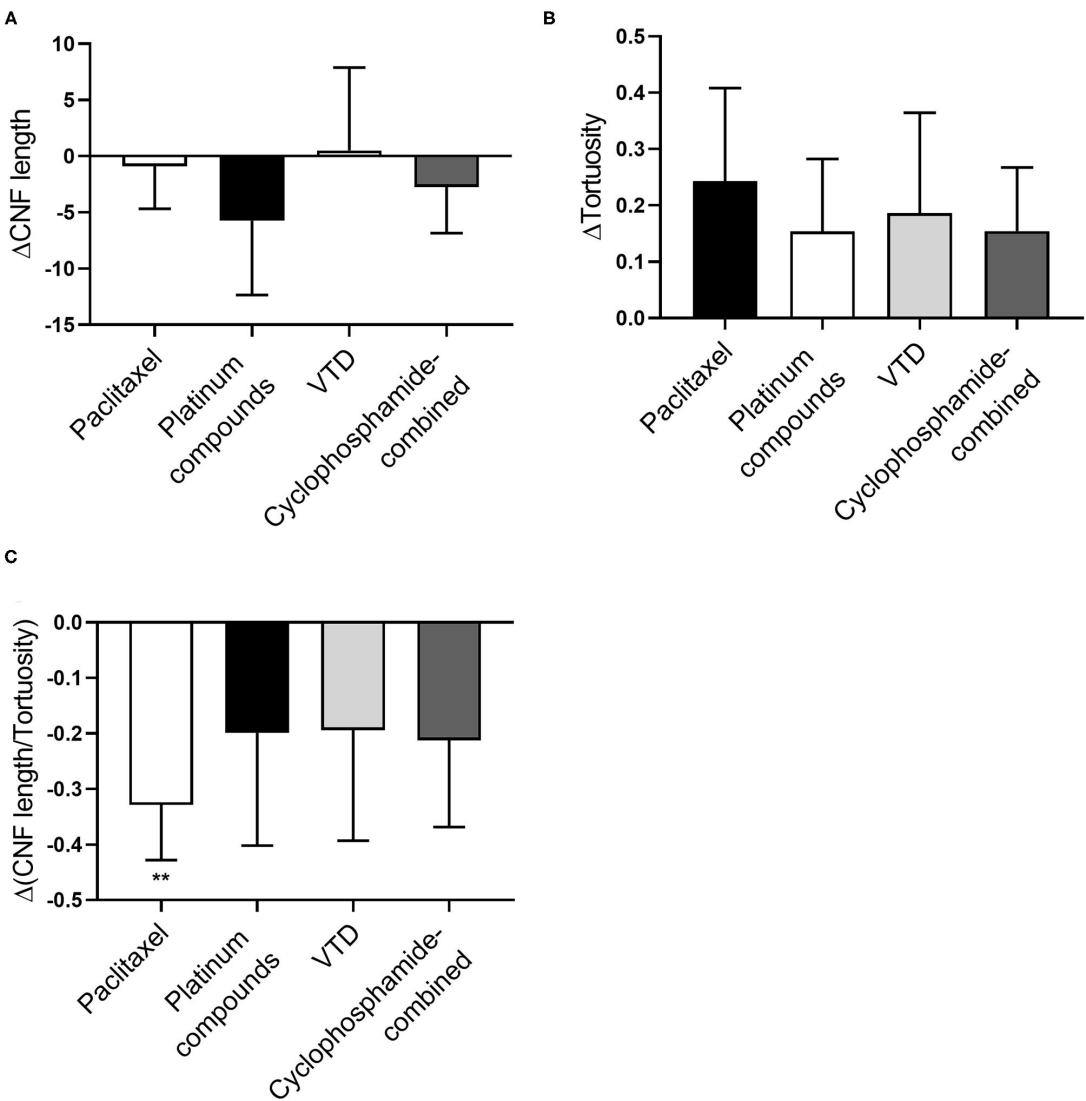
Analysis of corneal nerve parameters per drug group in a large cohort of patients. **(A)** Average ΔCNF length calculated before and after the different therapies. Paclitaxel *n* = 36 patients, Platinum compounds *n* = 12 patients, VTD *n* = 11 patients, Cyclophosphamide-combined *n* = 15 patients. **(B)** Average ΔTortuosity calculated before and after the different therapies. Paclitaxel *n* = 36 patients, Platinum compounds *n* = 12 patients, VTD *n* = 11 patients, Cyclophosphamide-combined *n* = 15 patients. **(C)** Average ΔCNF length—Tortuosity ratio calculated before and after the different therapies. Paclitaxel *n* = 36 patients, Platinum compounds *n* = 12 patients, VTD *n* = 11 patients, Cyclophosphamide-combined *n* = 15 patients. Statistical analysis by One sample *t*-test (**p < 0.01).

Following initial analysis on the entire group of patients, we considered for statistics the single drug groups of most neurotoxic agents. Even though patients receiving paclitaxel, platinum compounds and cyclophosphamide in combination with other drugs showed a decrease in CNFL, data did not reach statistical significance (Figure 2A). Similarly, tortuosity was increased in all groups, even though not significantly due to high variability (Figure 2B). CNFL/tortuosity ratio values were reduced in all drug groups, but it was only significant in paclitaxel group (*p* = 0.0023, Figure 2C).

### Nerve Morphology Quantification in the Epidermis and the Cornea

**Figure 3:**
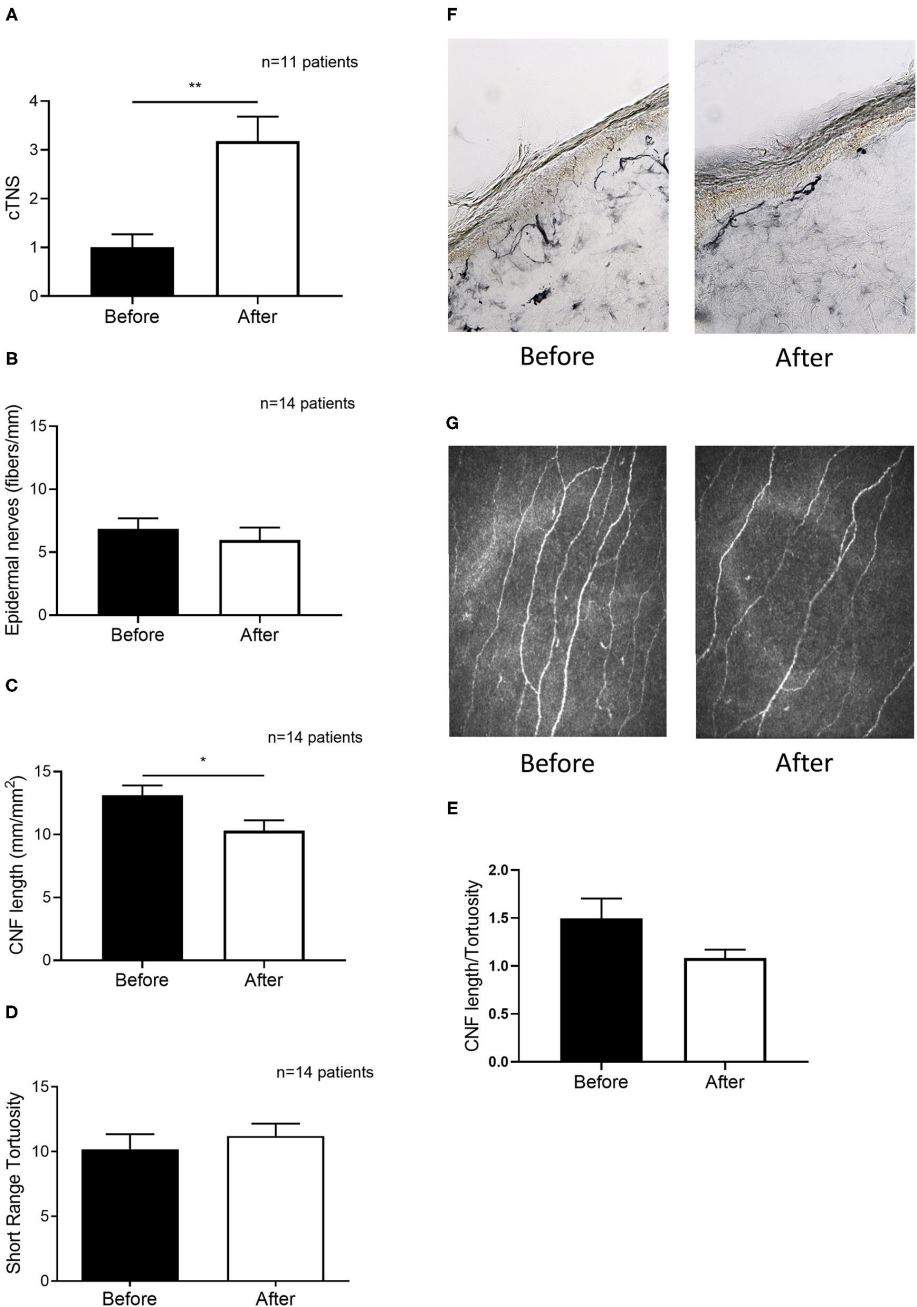
Chemotherapy-induced clinical worsening is paralleled by altered corneal but not epidermal nerve morphology in a subset of 14 patients. **(A)** Clinical TNS calculated in patients before and after the therapy. **(B)** Epidermal nerve fiber density from skin biopsies obtained in patients before and after the therapy. **(C)** Corneal Nerve Fiber length before and after therapy. **(D)** Corneal nerve tortuosity obtained by using the short-range tortuosity algorithm before and after therapy. **(E)** Corneal Nerve Fiber length—tortuosity ratio before and after therapy. **(F)** A representative picture of nerve fiber staining with PGP 9.5 **(G)** representative pictures of *in vivo* corneal confocal microscopy before and after therapy, which shows a significant reduction of corneal nerve fiber density. Histograms represent mean values ± SEM; Statistical analysis by Mann-Whitney non-parametric test (*p < 0.05, **p < 0.01).

A subgroup of 14 patients underwent both epidermal and corneal nerve examination. Patients' demographics are shown in Table 2. Clinical grading of neuropathy worsened significantly after chemotherapy initiation (+69% cTNS; *p* = 0.0018, Figure 3A). Yet, no changes in epidermal nerve density were detected (*p* = 0.4618, Figures 3B,F). Interestingly, however, corneal nerve density was significantly reduced (−22%, *p* = 0.0457, Figures 3C,G). No significant differences were observed in corneal nerve tortuosity before and after chemotherapy (*p* = 0.6347, Figure 3D). A reduction was observed, although not significant, in the CNF density/tortuosity ratio following chemotherapy (*p* = 0.1251, Figure 3E).

**Table 2:** Demographics of the subgroup of 14 patients.

***n*°**	**Cancer type**	**Age**	**Chemotherapy**
1	Amyloidosis	50	Cyclophosphamide-Bortezomib-Dexamethasone + Velcade-Dexamethasone + Lenalidomide-Dexamethasone
2	Multiple myeloma	55	Velcade-Thalidomide-Dexamethasone + High-dose Cyclophosphamide + Melphalan 200 mg/m^2^
3	Multiple myeloma	61	Dexamethasone + Velcade-Thalidomide-Dexamethasone + Cyclophosphamide + Melphalan 200 mg/m^2^
4	Breast cancer	50	Paclitaxel + Doxorubicin and Cyclophosphamide-Methotrexate-Fluorouracil (5FU)
5	Pancreas cancer	47	Paclitaxel albumin-Gemcitabin-Cisplatin
6	Colon cancer	73	Oxaliplatin and capecitabine
7	Bladder cancer	76	Low-dose Cisplatin
8	Multiple myeloma	65	Velcade-Thalidomide-Dexamethasone + Cyclophosphamide + Melphalan 200 mg/m^2^
9	Breast cancer	52	Paclitaxel + Doxorubicin and Cyclophosphamide-Methotrexate-Fluorouracil (5FU)
10	Amyloidosis	73	Melphalan 200 mg/m2 + Dexamethasone + Prednisone + Endoxan + lenalidomide plus dexamethasone + Cyclophosphamide-Prednisone + Cyclophosphamide-Dexamethasone + Cyclophosphamide/Docetaxel-Pirarubicin-ifosfamide-Velcade
11	Multiple myeloma	45	Velcade-Thalidomide-Dexamethasone + Cyclophosphamide + Melphalan 200 mg/m^2^
12	Tonsil cancer	70	Cisplatin
13	Multiple myeloma	39	Velcade-Thalidomide-Dexamethasone + Melphalan 200 mg/m^2^
14	Breast cancer	57	Paclitaxel + Doxorubicyn and Cyclophosphamide + Methotrexate + Fluorouracil (5FU)

## Discussion

The cornea is the most densely innervated tissue of the body; therefore, it is ideally suited to study nerve pathology. We previously showed that corneal and epidermal nerve density show good correlation in an animal model of paclitaxel-induced neuropathy (23). Recently, *in vivo* corneal confocal microscopy (IVCM) has gained popularity because it is an *in vivo*, non-invasive, repeatable imaging technique that allows detailed examination of corneal nerves (24, 25). In fact, IVCM has been used to evaluate peripheral nerve damage following chemotherapy with neurotoxic agents such as oxaliplatin, although no association with epidermal nerve density was provided (26–28), and paclitaxel (28).

Emerging evidence demonstrates the potential diagnostic and prognostic power of IVCM in peripheral neuropathies (12–14); still the clinical utility of this technique is matter of debate. For instance, it has been suggested that IVCM is not useful when CIPN is mild to moderate, at least when corneal nerve length and density are measured (29).

Here, we provide for the first time a comprehensive study, analyzing patients with several different types of cancer and undergoing different pharmacological treatments (paclitaxel, platin, VTD, and cyclophosphamide-combined). We show that neuropathy clinical grading (cTNS) worsens following chemotherapy, supporting that these medications are neurotoxic. In particular, ΔTNS was significantly affected after treatment with paclitaxel, platinum compounds and cyclophosphamide in combination with other drugs. Unexpectedly, the peripheral neuropathy severity of cyclophosphamide group was similar to the other more neurotoxic drugs. This could be a consequence of the combined therapy with other neurotoxic compound (i.e., paclitaxel or platinum compounds).

Interestingly, epidermal nerve length was not reduced in the subgroup where it was measured. This was unexpected and may be a consequence of the limited sample of patients who accepted to undergo biopsy, and/or to the inter- and intra-subject variability of such a marker (30). It is worth noting that IVCM showed an increased sensitivity in detecting peripheral neuropathy than quantification of epithelial nerves in our cohort. Indeed, tortuosity was increased in all drug groups, even though not significantly, and similarly CNFL was reduced in paclitaxel, platinum compounds and cyclophosphamide groups. Moreover, IVCM has the advantage of being non-invasive and repeatable, which makes it a more suitable option in CIPN patients.

Since reduced density and increased tortuosity of corneal nerves are hallmarks of peripheral neuropathy (12–14, 21), we decided to combine both parameters by calculating their ratio. Interestingly, the CNF density/tortuosity ratio, previously used to successfully diagnose neuropathy in diabetic patients (22), was more effective in detecting the alterations in corneal nerves induced by chemotherapy than the two parameters separately. In particular, paclitaxel group, which was the therapy administered more frequently, showed more severe nerve loss, and correlated with the cumulative dose received from the patient. One cross sectional study (29) did not find nerve parameters useful to detect peripheral neuropathy. Our prospective study analyzed corneal nerve morphology before and after treatment, which we believe brings advantages over cross-sectional design, because high corneal nerve density variability in humans could impact the ability to detect between group differences. Other authors have shown that CNF length was increased following chemotherapy in a small cohort of patients. However, the study only included upper gastrointestinal cancer patients, a small number of which attending the follow-up, and considered a small sample (3 pictures per eye), which were manually counted (31).

Finally, we observed the CNF density/tortuosity ratio was inversely correlated with the number of chemotherapy cycles, which suggests that it can capture cumulative drug toxicity.

While further studies are needed to elucidate which morphological parameters better parallel clinical signs and symptoms, our results suggest that measurement of corneal nerve density and tortuosity by IVCM shows promise for the detection of peripheral neuropathy in patients undergoing neurotoxic chemotherapy.

## Data Availability Statement

The raw data supporting the conclusions of this article will be made available by the authors, without undue reservation.

## Ethics Statement

The studies involving human participants were reviewed and approved by the Comitato Etico Istituto Scientifico Ospedale San Raffaele. The patients/participants provided their written informed consent to participate in this study.

## Author Contributions

NR, FB, RL, and GF: drafting the work and revising it critically for important intellectual content. FB, RL, PF, MB, FS, AR, and IL: organized the database, analysis of data, and performed the statistical analysis. GF, MR, TD, IL, AQ, NR, FA, PR, and MM: acquisition of data. GF: provide final approval for publication of the content. All authors contributed to manuscript revision, read, and approved the submitted version.

## Funding

This study has been funded by the Italian Department of Health (grant code: GR-2010-2319274).

## Conflict of Interest

AR was employed by Resono Ophthalmic srl. The remaining authors declare that the research was conducted in the absence of any commercial or financial relationships that could be construed as a potential conflict of interest.

## Publisher's Note

All claims expressed in this article are solely those of the authors and do not necessarily represent those of their affiliated organizations, or those of the publisher, the editors and the reviewers. Any product that may be evaluated in this article, or claim that may be made by its manufacturer, is not guaranteed or endorsed by the publisher.
